# Cyclodextrins: Advances in Chemistry, Toxicology, and Multifaceted Applications

**DOI:** 10.3390/molecules29225319

**Published:** 2024-11-12

**Authors:** Adina Magdalena Musuc

**Affiliations:** Institute of Physical Chemistry–Ilie Murgulescu, Romanian Academy, 202 Spl. Independentei, 060021 Bucharest, Romania; amusuc@icf.ro

**Keywords:** cyclodextrins, inclusion complexes, drug delivery, pharmacokinetics, nanomaterials, environmental remediation, supramolecular chemistry, smart materials

## Abstract

Cyclodextrins (CDs) have garnered significant attention in various scientific and industrial fields due to their unique ability to form inclusion complexes with a wide range of guest molecules. This review comprehensively explores the latest advancements in cyclodextrin chemistry, focusing on the synthesis and characterization of cyclodextrin derivatives and their inclusion complexes. This review examines the biological activities of cyclodextrins, highlighting their pharmacological properties and pharmacokinetics, and discussing their promising applications in drug delivery systems. Furthermore, the industrial utilization of cyclodextrins, including their role in nanomaterials and nanostructured coatings, as well as their potential in environmental remediation, are explored. The present research also addresses the critical aspect of toxicity, particularly concerning cyclodextrin inclusion complexes, providing an overview of the current understanding and safety considerations. Through a multidisciplinary approach, the aim is to present a complete view of cyclodextrins, underscoring their versatility and impact across various domains.

## 1. Introduction

**Figure 1:**
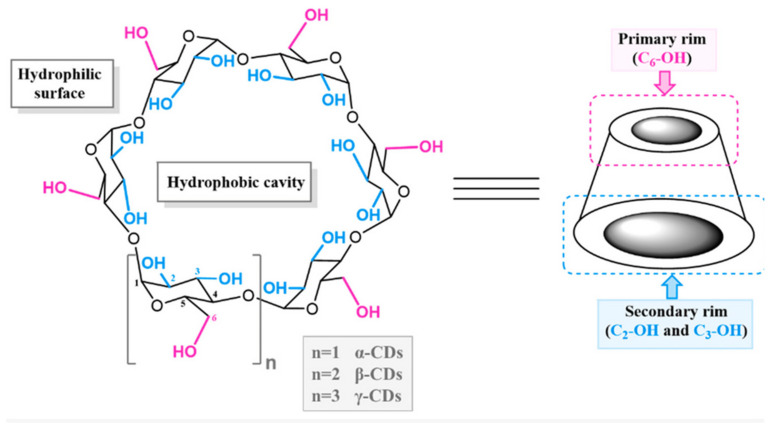
The general structure of CD (the figure represents the structure of the first three CDs: α-CD, β-CD, and γ-CD) [3].

Cyclodextrins (CDs) are a family of cyclic oligosaccharides composed of D-glucose subunits linked by α-(1→4) glycosidic bonds [1,2]. These macrocyclic oligomers of glucose exhibit a toroidal three-dimensional (3D) structure. The dimensions of this cavity vary based on the number of D-glucose units. Among these, the most common types are α-, β-, and γ-cyclodextrins, which are composed of six, seven, and eight glucopyranose units, respectively (Figure 1).

Their ability to selectively encapsulate molecules, thereby altering the solubility, stability, and bioavailability of guest compounds, has made cyclodextrins an indispensable tool in many scientific and industrial fields. While α-, β-, and γ-cyclodextrins are the most commonly studied and used in industry, large-ring cyclodextrins (LR-CDs) consist of more than 8 glucose units, typically ranging from 9 to 12 or even more glucose units in the ring [4]. Examples include δ-CD (9 units), ε-CD (10 units), and others with even larger ring sizes. This expanded cavity allows them to encapsulate larger guest molecules, making them suitable for inclusion complexes with macromolecules or compounds with bulkier structures. The size of the CD cavity determines the range of guest molecules that can be accommodated, with α-CD, β-CD, and γ-CD having progressively larger cavities suitable for different-sized guests (Table 1).

CDs are derived from starch through enzymatic conversion and are characterized by their unique molecular structure—a hydrophobic cavity and a hydrophilic exterior (Figure 1) [1,2,3]. This dual nature enables CDs to form inclusion, making them highly versatile in various applications, including pharmaceuticals, biotechnology, environmental science, and material engineering [6,7,8].

Since their discovery by Villiers in 1891 [9] and the subsequent identification of their molecular structure by Schardinger in the early 20th century [10,11], CDs have transitioned from being chemical curiosities to crucial functional molecules with diverse applications. The versatility of CDs stems not only from their inherent structural properties but also from the wide range of chemical modifications possible, leading to the creation of numerous cyclodextrin derivatives with tailored functionalities.

The ability of cyclodextrins to enhance the solubility, stability, and bioavailability of drugs has been a driving force behind their widespread use in drug delivery systems [12,13]. The pharmaceutical industry has particularly benefited from cyclodextrin chemistry, where their inclusion complexes have improved the therapeutic efficacy of numerous active pharmaceutical ingredients. Additionally, cyclodextrin derivatives have expanded the scope of these applications, offering tailored properties for specific needs.

Beyond the pharmaceutical domain, cyclodextrins have found significant roles in various industrial applications. Their incorporation into nano-sponges, nanomaterials, and nanostructured coatings has opened new avenues in material science, providing enhanced functionality and performance in sectors such as food, cosmetics, and textiles [14,15]. Moreover, their potential in environmental remediation, through the sequestration of pollutants and toxic substances, has emerged as a promising area of research [16].

The chemistry of cyclodextrins has rapidly advanced in recent years, allowing for precise chemical modifications that adapt their inclusion capabilities and optimize their functionality for specific uses. Cyclodextrins can be chemically altered through the addition of various substituents to the primary or secondary hydroxyl groups, enhancing their solubility, binding affinity, or selectivity for guest molecules [17]:

(i) Methylation involves the substitution of hydroxyl groups on the cyclodextrin molecule with methyl groups, resulting in derivatives such as methyl-β-cyclodextrin (M-β-CD). This modification enhances the hydrophobic character of cyclodextrin, thereby improving its solubility in organic solvents and increasing its ability to solubilize hydrophobic guest molecules. Dimethyl-beta-cyclodextrin (DIMEB) is characterized by the substitution of two hydroxyl groups on each glucose unit with methyl groups. DIMEB is known for its enhanced hydrophobicity and reduced toxicity compared to native β-cyclodextrin, making it suitable for applications in drug delivery and the encapsulation of hydrophobic drugs. DIMEB is particularly effective in forming inclusion complexes with poorly soluble compounds, enhancing their solubility and bioavailability. Randomly methylated beta-cyclodextrin (RAMEB) is produced through the random methylation of β-cyclodextrin, resulting in a mixture of substitution patterns. This randomness provides a balance of hydrophilicity and hydrophobicity, making RAMEB highly versatile in various applications, including pharmaceutical formulations and food industry applications. The variability in substitution can lead to unique interaction profiles with guest molecules, allowing for tailored complexation properties.
(ii) Hydroxypropylation involves the introduction of hydroxypropyl groups to the cyclodextrin structure. Hydroxypropyl-β-cyclodextrin (HP-β-CD) is a notable example, offering improved water solubility and biocompatibility compared to its native counterparts. This modification not only increases the solubility of HP-β-CD but also enhances its ability to form inclusion complexes with various pharmaceuticals, thus facilitating controlled release and targeted drug delivery applications.
(iii) Acylation modifies cyclodextrins by introducing acyl groups, which can alter the solubility and lipophilicity of the derivatives. Acylated cyclodextrins can be tailored for specific applications in drug delivery systems, providing better drug stabilization and release profiles.
(iv) Sulfation and phosphorylation introduce charged groups onto the cyclodextrin backbone, increasing hydrophilicity and enhancing interaction with biological systems. Functionalization techniques allow for the attachment of various functional groups (e.g., amino, carboxylic, or thiol groups) to cyclodextrins. This customization can fit the interaction properties of CDs, enhancing their performance in applications, such as chiral separation, drug delivery, and targeted therapies.

These modifications have expanded the potential of CDs, particularly in advanced biomedical applications, such as gene and drug delivery, tissue engineering, and nanotechnology [18,19]. By introducing functional groups, researchers can create derivatives that respond to environmental stimuli, making cyclodextrins valuable in developing smart drug delivery systems. Additionally, modified CDs exhibit improved binding with biomolecules, enabling their use in diagnostic and therapeutic applications [20].

However, the widespread use of cyclodextrins also raises concerns regarding their safety and toxicity, particularly in the context of inclusion complexes. Understanding the toxicological profile of cyclodextrins and their derivatives is crucial for ensuring their safe application across different industries. Cyclodextrins are generally regarded as safe, with low toxicity profiles and minimal side effects, and they have further gained popularity, particularly in the pharmaceutical and food industries. However, concerns about the safety of high doses of cyclodextrins, particularly β-CD, have been raised, as prolonged exposure or large amounts can lead to gastrointestinal and renal issues [21]. As such, ongoing research aims to thoroughly understand the toxicological profiles of different types of cyclodextrins, as well as their biodegradability and potential environmental impact.

When administered orally, CDs tend to exhibit low toxicity, primarily due to their poor absorption in the gastrointestinal tract. Their limited systemic exposure when administered orally contributes to their favorable safety profile, making them suitable for use in formulations, such as tablets, capsules, and oral suspensions. For example, α-cyclodextrin has been used as a dietary fiber supplement [22], and derivatives of β-cyclodextrin are common excipients in oral formulations, as they can enhance the solubility of poorly water-soluble drugs without causing notable adverse effects [23,24]. However, the safety of cyclodextrins can change dramatically when they are administered through parenteral routes, such as intravenous (IV) or intramuscular (IM) injections. In these cases, cyclodextrins have direct access to systemic circulation, and their impact on organs, such as the kidneys, becomes more relevant. For example, β-cyclodextrin and its derivatives have been associated with nephrotoxicity when used at high doses or over extended periods [25]. The potential for accumulation in the renal tubules can lead to renal impairment or kidney damage, as has been observed in some animal studies. This risk is particularly pronounced with highly substituted derivatives, such as methyl-β-cyclodextrin, which can interact with cell membranes and induce toxicity [25]. Nevertheless, the dosage and frequency of administration remain critical factors, as even derivatives with improved safety can pose risks at higher concentrations. Inhalation is another route where the safety profile of cyclodextrins must be considered carefully. Although inhalable CDs, such as HP-β-CD (hydroxypropyl-β-cyclodextrin), have been investigated for enhancing the delivery of pulmonary drugs, their potential to cause pulmonary irritation or inflammation requires a thorough assessment [26]. Overall, the route of administration is a critical determinant in the safety evaluation of cyclodextrins, influencing factors such as bioavailability, systemic exposure, and organ-specific toxicity. The careful selection of cyclodextrin type, derivative, and dosage can thus enable the effective use of these versatile molecules across a wide range of pharmaceutical applications, from oral supplements to advanced parenteral therapies.

Despite these findings, the potential applications of CDs continue to expand. In drug delivery, cyclodextrins are being explored as carriers for anticancer agents, antibiotics, and antiviral drugs, improving their therapeutic efficacy while reducing toxicity. Their use in cosmetics has evolved beyond stabilizers for fragrances, with new formulations incorporating CDs to enhance skin penetration and provide sustained release of active ingredients. Environmental science has also seen growing interest in using CDs for pollutant remediation, with CDs acting as agents that can trap toxic chemicals, heavy metals, and other pollutants from water and soil. Additionally, in nanotechnology, cyclodextrin-based systems are being developed for constructing nanocarriers, sensors, and other nanodevices, capitalizing on their versatile and adaptable molecular framework.

The novelty of this review lies in its complete and updated approach to the current state of cyclodextrin chemistry and toxicology, with a focus on their inclusion complexes, derivatives, biological activities, and industrial applications. Additionally, it will examine the emerging field of cyclodextrin-based nanomaterials and their implications for environmental remediation. It will explore the latest innovations in modifying cyclodextrins for improved performance, discuss safety considerations based on current toxicological studies, and examine how cyclodextrins are being used in emerging fields, such as biotechnology, nanomedicine, and environmental science. As research into cyclodextrins continues to evolve, these molecules are poised to make even greater contributions to science and industry, offering new opportunities for innovation, particularly in solving complex challenges related to drug delivery, environmental sustainability, and material science. By synthesizing the latest research findings, this article seeks to highlight the multifaceted nature of cyclodextrins and their potential to drive innovation across a wide range of scientific and industrial disciplines.

## 2. Cyclodextrin Complexation Chemistry

Cyclodextrin complexation chemistry covers the synthesis, structural modification, and complexation behavior of CDs. The parent cyclodextrins (α-, β-, and γ-CD) can be chemically modified to enhance their solubility, stability, or inclusion capacity, giving rise to a vast array of cyclodextrin derivatives. These modifications include the introduction of hydrophilic, hydrophobic, or ionic groups, which can significantly alter the physicochemical properties of the resulting CDs (Table 2).

**Figure 2:**
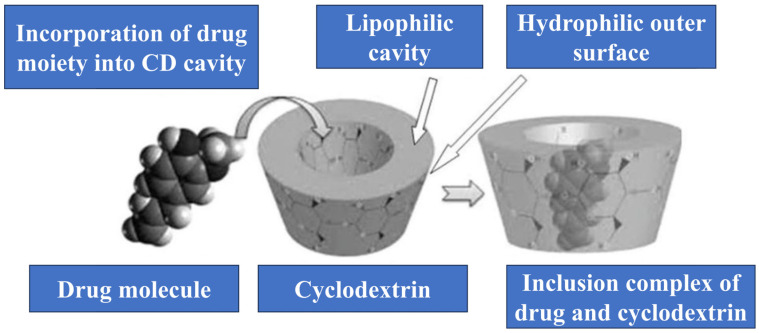
Schematic representation of the inclusion mechanism of a drug into the cyclodextrin cavity (adapted from [5]).

The creation of derivatives, such as hydroxypropyl-β-cyclodextrin and methyl-β-cyclodextrin, has expanded the application potential of CDs, particularly in drug delivery, where they can be used to improve the bioavailability and therapeutic index of drugs [32]. The inclusion complex formation is the basis of cyclodextrin supramolecular chemistry. The ability of CDs to host guest molecules within their hydrophobic cavity allows them to stabilize and solubilize otherwise poorly soluble compounds (Figure 2). This property has been exploited extensively in the pharmaceutical industry, where CDs are used to enhance the solubility and stability of active pharmaceutical ingredients (APIs). Furthermore, the supramolecular chemistry of CDs, which involves non-covalent interactions, such as hydrogen bonding, van der Waals forces, and hydrophobic interactions, plays a critical role in the formation and stability of these inclusion complexes.

### 2.1. Structural Modifications and Derivative Synthesis

The chemical modification of cyclodextrins is a critical area of research aimed at enhancing their solubility, stability, and inclusion complexation properties. Derivatives such as hydroxypropyl-β-cyclodextrin, methyl-β-cyclodextrin, and sulfobutylether-β-cyclodextrin are widely used in pharmaceuticals due to their improved aqueous solubility. Hydroxypropyl-β-cyclodextrin (HP-β-CD) is broadly used in pharmaceuticals due to its improved solubility and safety profile. It is utilized in the formulation of intravenous drugs, such as itraconazole, to enhance solubility [33]. The synthesis of these derivatives typically involves the reaction of native CDs with reagents that introduce functional groups to the hydroxyl groups on the glucose units. Cyclodextrin functionalization is heavily influenced by the differing reactivity of its hydroxyl groups, specifically the primary hydroxyl groups located on the narrower rim of the molecule and the secondary hydroxyl groups situated on the wider rim. The primary hydroxyl groups at the 6-position are generally more reactive due to their exposure and spatial accessibility, making them more amenable to modifications, such as methylation or sulfation, which can significantly enhance solubility and improve binding affinities. In contrast, the secondary hydroxyl groups at the 2- and 3-positions often require selective protection strategies during functionalization processes due to their steric interference and relatively lower reactivity. By strategically modifying these hydroxyl groups, researchers can design cyclodextrin derivatives with tailored properties suited for specific applications, including drug delivery, environmental remediation, and catalytic processes. For instance, derivatization at the secondary hydroxyl positions can create cyclodextrins with enhanced hydrophilicity or ionizable functional groups, enabling improved interaction with target molecules. This nuanced approach to functionalization highlights the chemical versatility of cyclodextrins and expands their applicability across a broad spectrum of scientific fields [34]. These modifications can either enhance the hydrophilicity of CDs, allowing them to be used in aqueous environments, or introduce hydrophobic moieties that improve the binding affinity for certain guest molecules. While unmodified cyclodextrins have significant potential in drug delivery, pre- and post-modifications of CDs have expanded their functionality, enabling the development of various innovative materials. The polyhydroxy framework of CDs allows them to react with a diverse array of reagents, producing water-soluble CD derivatives. By targeting specific hydroxyl groups—such as the primary 6-OH group or the secondary 2- and 3-OH groups—researchers can introduce various functional groups through chemical modifications, making these supramolecular structures suitable for drug delivery applications. Functional modifications of CDs can incorporate ionic, hydrophobic, or other active groups (e.g., azide, p-toluene sulfonyl, halogen, and sulfhydryl groups). These changes enhance their applicability, including use as gene delivery vectors or excipients that improve permeation [35,36].

One of the major advantages of cyclodextrin derivatives is their ability to selectively form inclusion complexes with a wide range of guest molecules, ranging from small organic molecules to large macromolecules. The choice of a derivative depends on the specific application, with some derivatives being more suitable for stabilizing volatile compounds, while others are better suited for enhancing the solubility of hydrophobic drugs. Additionally, the degree of substitution (i.e., the number of functional groups attached to the CD) plays a significant role in determining the inclusion complex’s stability and solubility.

### 2.2. Mechanisms of Inclusion Complex Formation

Inclusion complex formation between cyclodextrins and guest molecules is often governed by enthalpy–entropy compensation, a thermodynamic phenomenon where changes in the enthalpy (Δ*H*) are balanced by corresponding changes in entropy (Δ*S*). This balance plays a critical role in determining the stability and specificity of cyclodextrin inclusion complexes. Enthalpic contributions typically arise from interactions such as hydrogen bonding and van der Waals forces between the cyclodextrin cavity and the guest molecule, stabilizing the complex. Conversely, entropy changes are influenced by factors such as the release of water molecules from the hydrophobic cavity of cyclodextrin, which can favor complex formation due to the increase in system entropy. The balance between enthalpy and entropy is crucial, as it influences the overall Gibbs free energy (Δ*G*) of complexation, determining whether the formation of the inclusion complex is thermodynamically favorable. For example, a strongly exothermic enthalpy change may result in a more stable complex, even if it suffers a reduction in entropy. This compensation mechanism also helps to explain the selectivity of cyclodextrins for certain guest molecules, as optimal fits between the cavity and guest often result in the most favorable enthalpy–entropy balances. By understanding these thermodynamic properties, researchers can better predict and design cyclodextrin complexes with desired stability and specificity for targeted applications [37]. The hydrophobic cavity of the CD provides a suitable environment for the encapsulation of hydrophobic moieties of the guest, while the hydrophilic exterior ensures that the complex remains soluble in water. A crucial aspect of these complexes is the host-to-guest molar ratio, which can vary depending on the size, shape, and polarity of both the guest molecule and the cyclodextrin used. Commonly observed molar ratios include 1:1 (one guest molecule per one CD molecule), but 1:2 and 2:1 ratios can also occur depending on the interaction dynamics between the host and the guest. Determining the precise host–guest molar ratio in a cyclodextrin inclusion complex can be challenging, as it requires a deep understanding of the molecular interactions that govern the complexation process.

The inclusion complex of cyclodextrin with cholesterol has been studied extensively for its role in reducing cholesterol levels by increasing its solubility and bioavailability [38]. Curcumin, a poorly soluble natural compound, forms stable inclusion complexes with γ-cyclodextrin, significantly improving its solubility and bioavailability [39].

A representative example of the challenges involved in determining host–guest molar ratios is seen in the complexation of 17-β-estradiol with β-cyclodextrin. Studies have shown that this complex can exhibit different stoichiometric ratios, such as 1:1 and 1:2, depending on the concentration of the estradiol and β-cyclodextrin in the solution. The 1:1 complex is typically formed under conditions where the guest concentration is low, while the 1:2 complex may occur at higher guest concentrations or in specific solvent conditions that facilitate the binding of two cyclodextrin molecules to one guest molecule [40].

The process of complexation is influenced by factors such as the concentration of the CD and guest, the solvent environment, temperature, and pH. The inclusion process can be either spontaneous or facilitated by external factors, such as temperature or the presence of co-solvents. The molar ratios of CDs to guest molecules are critical in determining the efficiency of complexation. Generally, higher concentrations of either component can lead to a greater probability of complex formation, but beyond a certain point, the formation of aggregates or unbound guests may occur. This phenomenon necessitates careful optimization of the CD-to-guest ratio. The choice of solvent significantly influences complexation. CDs typically exhibit better solubility and complexation efficiency in polar solvents, such as water and alcohol. The polarity and dielectric constant of the solvent can modulate the interaction strength between the CD and the guest. Temperature plays a dual role in complexation. Increased temperature can enhance the solubility of the guest and the kinetic energy of molecules, potentially favoring complex formation. However, excessive temperatures may destabilize the complex by disrupting intermolecular interactions. Similarly, pH can affect the ionization state of both CDs and guest molecules, influencing their ability to form stable complexes. For instance, the pH can alter the charge state of certain functional groups on the guest, impacting its affinity for the hydrophobic cavity of the CD. By understanding the complexation process and the factors that influence it, researchers can better harness the unique properties of cyclodextrins in various applications. Techniques such as phase solubility analysis, NMR spectroscopy, and X-ray crystallography are commonly used to study the structure and stability of inclusion complexes. These methods provide insights into the orientation of the guest within the CD cavity, the strength of the complexation, and the thermodynamics involved.

Among the array of analytical techniques used, solid-state nuclear magnetic resonance (ssNMR) spectroscopy stands out for its ability to provide detailed insights into the molecular interactions within cyclodextrin inclusion complexes. Unlike solution-state NMR, ssNMR can probe the solid-state characteristics of cyclodextrin complexes, making it particularly valuable for analyzing complexation states, molecular orientation, and structural dynamics in powders, crystals, and amorphous materials. Studies have emphasized the application of ssNMR in the field of cyclodextrin research, demonstrating its utility in determining binding affinities, guest–host orientation, and structural changes during complexation. These studies provide an extensive overview of how ssNMR techniques, such as cross-polarization magic angle spinning (CP/MAS) and 2D NMR correlation methods, can be extended to gain deeper insights into the structural properties of cyclodextrin systems [41].

### 2.3. Cyclodextrin-Based Nano-Sponges

Cyclodextrin-based nano-sponges represent a novel and promising application of cyclodextrin derivatives, particularly in the field of drug delivery systems [42]. These materials are three-dimensional, cross-linked polymeric networks that are synthesized by combining cyclodextrins (CDs) with cross-linking agents, resulting in highly porous structures. The porous nature of these nano-sponges allows them to encapsulate a wide range of molecules, making them ideal for applications in drug loading, sustained release, and targeted drug delivery. The basic structure of cyclodextrin-based nano-sponges involves a network of cyclodextrins cross-linked with various agents, such as diphenyl carbonate, pyromellitic dianhydride, or epichlorohydrin. This cross-linking creates a rigid matrix with nano-sized cavities that can encapsulate hydrophobic drugs within the inner cavity of the cyclodextrin, as well as hydrophilic drugs in the polymeric network itself [43]. The synthesis process can be tailored to control the porosity, particle size, and surface properties of the nano-sponges, enabling customization for specific therapeutic needs. One commonly used method for the preparation of nano-sponges involves polycondensation, where CDs are mixed with a cross-linker under controlled heating and stirring conditions to form a highly cross-linked network. Alternatively, emulsion-based techniques are also used to produce nano-sponges with well-defined particle sizes, making them suitable for parenteral and oral drug delivery applications. The introduction of cyclodextrin-based nano-sponges into drug delivery systems provides several advantages over conventional cyclodextrin complexes: (i) First, the higher drug loading capacity of nano-sponges allows for the incorporation of larger doses of active pharmaceutical ingredients (APIs), making them more effective for high-dose therapies. (ii) Second, the sustained-release profile of nano-sponges can help maintain steady drug levels in the body, reducing the peaks and troughs associated with frequent dosing. (iii) Third, their ability to be surface-modified allows for the customization of nano-sponges for site-specific delivery, addressing the limitations of non-targeted therapies. Moreover, the flexibility in the synthesis of nano-sponges enables the creation of biocompatible and biodegradable materials, which is particularly important for reducing the environmental impact and ensuring safe metabolization within the body. These properties have made cyclodextrin-based nano-sponges a subject of interest not only in pharmaceutical research but also in cosmetics and nutraceuticals [44].

## 3. Applications of Cyclodextrin Inclusion Complexes

Cyclodextrin inclusion complexes have become invaluable in a wide range of industries due to their ability to encapsulate guest molecules, significantly altering and improving the physical and chemical properties of these substances. This unique characteristic of cyclodextrins has led to their widespread adoption, particularly in the pharmaceutical industry, where they play a crucial role in enhancing the solubility, stability, and bioavailability of poorly water-soluble drugs. Furthermore, CDs can modulate the release profile of drugs, enabling controlled or sustained release, which is crucial for maintaining therapeutic drug levels over an extended period [45]. Piroxicam, a nonsteroidal anti-inflammatory drug (NSAID), forms an inclusion complex with β-CD, leading to enhanced solubility and reduced gastrointestinal irritation [46].

Many active pharmaceutical ingredients (APIs) suffer from low aqueous solubility, which limits their absorption and effectiveness. By forming inclusion complexes with cyclodextrins, these drugs can be solubilized in water, improving their dissolution rates and increasing their bioavailability. This has been especially beneficial for drugs that would otherwise have limited therapeutic effects due to their poor solubility. In addition to enhancing solubility, cyclodextrins can also protect drugs from degradation, whether by light, heat, or oxidation, which can occur during storage or upon administration [47]. This protective function not only prolongs the shelf life of pharmaceutical products but also ensures the delivery of the active ingredient in its most effective form. Furthermore, cyclodextrin inclusion complexes can mask undesirable properties of certain drugs, such as unpleasant tastes or odors, making them more palatable and thus improving patient compliance, particularly in oral formulations [48]. Cyclodextrins are also employed to control the release of drugs, enabling sustained or targeted delivery. By modulating the rate at which the drug is released from the complex, it is possible to maintain therapeutic drug levels in the body over a longer period, reducing the need for frequent dosing and improving treatment outcomes [49].

Cyclodextrins have also emerged as highly effective chiral selectors in chromatographic techniques and other separation methods due to their unique structural characteristics and ability to form inclusion complexes with chiral compounds [50]. The ability of cyclodextrins to selectively interact with enantiomers is primarily attributed to their hydrophobic cavity, which provides a suitable environment for accommodating various guest molecules. When enantiomers are introduced to a solution containing cyclodextrins, they can form host–guest complexes based on their steric and electronic properties. The differential affinities of the enantiomers for the cyclodextrin cavity arise from factors such as size, shape, and functional group orientation. As a result, one enantiomer may bind more strongly to the cyclodextrin than its counterpart, leading to effective separation. For example, β-cyclodextrin is commonly used in chiral chromatography due to its ability to discriminate between enantiomers through hydrogen bonding, van der Waals forces, and hydrophobic interactions [18]. By utilizing chiral-derivatized cyclodextrins, such as those modified with polar functional groups, researchers can enhance selectivity and improve resolution during enantioseparation processes. The advantages of utilizing cyclodextrins as chiral selectors in enantioseparation processes are complex, as outlined in the following: (i) Cyclodextrins can selectively bind to specific enantiomers, enhancing resolution and sensitivity in chiral analyses. (ii) The structural diversity of cyclodextrins allows for modifications that can optimize interactions with a wide range of chiral compounds. (iii) Cyclodextrins can operate under mild conditions, reducing the likelihood of degradation or alteration of sensitive chiral compounds. (iv) Cyclodextrins are derived from natural sources and are biodegradable, making them more environmentally sustainable compared to some traditional chiral selectors.

Beyond the pharmaceutical industry, cyclodextrin inclusion complexes have found extensive applications in the food and beverage industry to stabilize flavors, fragrances, and active ingredients. Here, they are primarily used to stabilize volatile or sensitive compounds, such as flavors, colors, vitamins, and essential oils, that would otherwise degrade or evaporate during processing and storage. Cyclodextrins encapsulate these compounds, protecting them from external factors, such as oxidation, light, and temperature changes, which can lead to a loss of potency or effectiveness. In these industries, CDs also help prevent the volatilization or degradation of sensitive compounds, thereby extending the shelf life of products. Essential oils (e.g., peppermint oil) are encapsulated in CDs for use in food preservation and flavor enhancement, protecting the oils from volatilization and degradation [51]. For example, in the stabilization of flavors, cyclodextrins can trap volatile molecules, preventing them from evaporating and ensuring that food products retain their intended taste and aroma even after extended storage. This not only improves the sensory qualities of the food but also enhances its overall quality and consumer appeal. Similarly, in the preservation of vitamins and antioxidants, cyclodextrins help to maintain their stability and bioavailability, ensuring that nutritional supplements or fortified foods deliver their intended health benefits. Cyclodextrin complexes are also used in food packaging, where they can trap and neutralize unpleasant odors or gases, further extending the shelf life of perishable products [15,52]. Cyclodextrins can form inclusion complexes with various molecules, such as pesticides, toxins, and food preservatives, enhancing the sensitivity and specificity of analytical methods. Many pesticides and toxins are hydrophobic and poorly soluble in water. When these compounds form inclusion complexes with cyclodextrins, their solubility is significantly enhanced, leading to better detection limits in analytical methods. Cyclodextrins can selectively bind to target molecules, reducing the background noise from other components in a sample. The enhanced sensitivity and specificity from cyclodextrin inclusion complexes are particularly beneficial in chromatographic techniques, such as HPLC and capillary electrophoresis (CE), where they facilitate the separation and quantification of target analytes at low concentrations. These complexes can be utilized in sensors and detection systems to accurately monitor the presence of harmful substances, ensuring food safety and quality control. Additionally, cyclodextrins aid in stabilizing sensitive analytes, improving the accuracy of food safety assessments.

In the cosmetics and personal care industry, cyclodextrin inclusion complexes have been employed to enhance the stability and efficacy of active ingredients, such as vitamins, peptides, and essential oils, which are commonly used in skincare, haircare, and other beauty products [13,53]. These active ingredients are often sensitive to environmental factors, such as light, air, and moisture, which can degrade their effectiveness over time. Additionally, cyclodextrins can provide a controlled release of fragrances, moisturizers, or therapeutic agents, allowing for longer-lasting effects on the skin or hair [54]. This has led to their use in formulations that aim to deliver prolonged hydration or gradual fragrance release, providing a more consistent and sustained user experience. Cyclodextrins are also used to reduce the irritation potential of certain active ingredients, making them more suitable for sensitive skin or delicate areas of the body.

Environmental science is another field where cyclodextrin inclusion complexes have proven to be valuable, particularly in the remediation of pollutants. Cyclodextrins can encapsulate and trap organic pollutants, heavy metals, and other contaminants, facilitating their removal from water and soil [55]. This encapsulation reduces the mobility and bioavailability of harmful substances, minimizing their environmental impact and making them easier to extract and neutralize. For instance, cyclodextrins have been used to remove persistent organic pollutants (POPs) from contaminated water sources, effectively reducing the toxicity and health risks associated with these substances [56]. The ability of cyclodextrins to form complexes with a wide range of contaminants makes them a versatile tool in environmental cleanup efforts, contributing to more sustainable and effective pollution control strategies. Moreover, cyclodextrins are biodegradable and environmentally friendly, further supporting their use in green chemistry and eco-friendly technologies [57].

By tailoring the size and chemical properties of the cyclodextrin complex, it is possible to achieve targeted delivery, where the therapeutic agent is released only in the presence of certain stimuli, such as pH changes, temperature shifts, or specific enzymes. This has significant implications for the development of smart drug delivery systems, particularly in cancer therapy, where precise targeting of tumor cells while minimizing damage to healthy tissues is critical. Cyclodextrins are also being incorporated into biosensors, where they enhance the sensitivity and selectivity of the sensors by facilitating the detection of specific molecules or biomarkers [58]. In diagnostics, cyclodextrin-based materials are being used to improve the accuracy and efficiency of tests for detecting diseases or monitoring health conditions.

### 3.1. Cyclodextrin in Pharmacology and Pharmacokinetics

The field of pharmacology is continually evolving, driven by the need for innovative strategies to enhance the efficacy and safety of therapeutic agents. One of the primary challenges in drug delivery is the poor solubility of many therapeutic agents, which affects their bioavailability and ultimately limits their clinical effectiveness. Additionally, cyclodextrins can influence pharmacokinetic profiles, allowing for better control over drug release rates, duration of action, and overall therapeutic effectiveness. By altering the pharmacokinetic properties of drugs, cyclodextrins can help optimize treatment regimens, especially for medications with a narrow therapeutic index. Despite the numerous advantages of cyclodextrins, the formulation of cyclodextrin–drug complexes presents several challenges, including potential toxicity and interactions with biological membranes. Understanding these complexities is crucial for the successful development and application of cyclodextrin-based formulations in clinical practice.

Subsequently, the role of cyclodextrins in pharmacology focuses on their ability to enhance drug solubility and bioavailability, modulate pharmacokinetics, and the challenges associated with their use in drug formulations. Through examining these aspects, this section aims to highlight the importance of cyclodextrins in improving therapeutic results and addressing the limitations of existing drug delivery systems.

#### 3.1.1. Enhancing Drug Solubility and Bioavailability

It is estimated that nearly 40% of drugs on the market face challenges related to low solubility, which significantly restricts their absorption and therapeutic effectiveness [59]. This is a critical issue since poor solubility can lead to inconsistent drug plasma levels, reduced efficacy, and an increase in side effects due to the need for higher doses.

Cyclodextrins attack this challenge through the formation of inclusion complexes. These complexes are formed when a drug molecule is encapsulated within the hydrophobic cavity of the cyclodextrin, increasing the apparent solubility of the drug in aqueous environments. For instance, griseofulvin, an antifungal agent traditionally known for its low solubility, shows significantly improved absorption when formulated as a complex with β-cyclodextrin [60]. Studies have demonstrated that this formulation enhances the drug’s bioavailability, resulting in more effective treatment results for fungal infections.

Furthermore, the bioavailability of a drug is not solely determined by its solubility—it also depends on its permeability across biological membranes. Cyclodextrins can influence the permeability of drugs by altering their distribution between aqueous and lipid phases. For example, cyclodextrins can enhance the permeation of lipophilic drugs, which would typically have poor absorption in the gastrointestinal tract [61]. By keeping these lipophilic compounds in a solubilized state, cyclodextrins facilitate their absorption through the lipid-rich membranes of the gastrointestinal tract. Ibuprofen, a widely used nonsteroidal anti-inflammatory drug (NSAID), illustrates this point well. When formulated with cyclodextrins, ibuprofen demonstrates increased absorption rates. After an initial rapid release within the first 5 min, ibuprofen is released more quickly from both β-cyclodextrin (β-CD)/ibuprofen (IBU) and β-CD/IBU/chitosan (CA) complexes until the 20 min mark, achieving dissolution rates of 93% and 98%, respectively [62]. Conversely, cyclodextrins can also limit drug permeability under specific conditions by forming stable complexes that are too large to pass through biological membranes [63]. This property is beneficial in developing controlled-release formulations, where a gradual release of the drug into the bloodstream is desirable.

#### 3.1.2. Modulating Drug Pharmacokinetics

The inclusion of drugs in cyclodextrin complexes significantly alters their pharmacokinetic profiles, which is crucial for optimizing therapeutic efficacy. By controlling the release rate of drugs from these complexes, cyclodextrins can prolong the duration of action, minimize the frequency of dosing, and reduce fluctuations in plasma drug concentrations. This ability is especially beneficial for drugs with a narrow therapeutic index, where maintaining consistent plasma levels is vital for both efficacy and safety. A notable example is digoxin, a cardiac glycoside used to treat heart conditions. When digoxin is formulated with β-cyclodextrin, its release can be controlled over a period ranging from 6 to 24 h, depending on the specific formulation and experimental conditions, resulting in extended therapeutic action [64]. This is particularly important in managing chronic conditions, as it reduces the need for frequent dosing and helps maintain stable plasma concentrations.

In addition to modulating release rates, cyclodextrins also provide a protective environment for drugs, shielding them from premature degradation due to enzymatic activity or metabolic processes. This protective effect is particularly important for drugs that are prone to rapid metabolism. Erythromycin, an antibiotic that is susceptible to degradation in acidic environments, benefits from being encapsulated in cyclodextrins [65]. This encapsulation ensures that the drug remains stable and effective upon oral administration, thus enhancing its therapeutic potential.

Moreover, cyclodextrins can influence the distribution of drugs throughout the body by altering their partitioning between blood and tissues. This capability can potentially reduce toxicity and minimize side effects associated with high drug concentrations in sensitive organs.

### 3.2. Challenges and Considerations in Cyclodextrin–Drug Formulations

Despite the numerous advantages that cyclodextrins offer in drug formulations, several challenges must be addressed to optimize their application in clinical settings. One of the primary concerns is the potential toxicity of cyclodextrins, particularly when used at high doses or over prolonged periods. The safety profile of cyclodextrin–drug complexes can vary significantly depending on the specific type of cyclodextrin employed, the route of administration, and the duration of exposure. β-Cyclodextrin has been associated with nephrotoxicity in some studies, particularly when administered in high doses [66]. This observation underscores the importance of carefully selecting the appropriate cyclodextrin derivative based on the specific therapeutic context and the patient population.

Another challenge is the interaction of cyclodextrins with biological membranes, which can potentially lead to membrane disruption and cytotoxicity. This issue is particularly relevant for methylated and other highly substituted cyclodextrins, which exhibit a higher affinity for lipid membranes. Such interactions can compromise cell membrane integrity, potentially leading to unwanted side effects. For instance, highly substituted cyclodextrins, such as hydroxypropyl-β-cyclodextrin, may demonstrate a stronger interaction with lipid membranes, raising concerns regarding cytotoxicity [63]. Table 3 highlights the recent applications of cyclodextrins in drug formulations. By leveraging the unique properties of cyclodextrins, researchers can enhance drug delivery systems, leading to improved and more effective treatments across various medical fields.

## 4. Industrial Applications of Cyclodextrin Inclusion Complexes in Advanced Drug Delivery Systems

Cyclodextrins have proven to be highly versatile molecules with applications extending far beyond pharmaceuticals and drug delivery. In recent years, the integration of CDs into emerging technologies, such as nanomaterials, nanostructured coatings, and environmental remediation, has gained increasing attention [74,75]. These applications influence the unique physicochemical properties of CDs, particularly their ability to encapsulate hydrophobic compounds, enhancing solubility, stability, and functionality in diverse settings.

Table 4 summarizes the wide range of industrial applications for cyclodextrin inclusion complexes, showcasing how they enhance solubility, stability, protection, and controlled release across various industries, such as pharmaceuticals, food, cosmetics, environmental science, and biotechnology.

Overall, the diverse applications of cyclodextrin inclusion complexes across various industries highlight their versatility and potential as multifunctional materials. Whether in pharmaceuticals, food, cosmetics, environmental science, or emerging technologies, cyclodextrins continue to provide innovative solutions that enhance product performance, safety, and sustainability. As research into cyclodextrin chemistry advances, new modifications and derivatives are being developed to expand their capabilities, offering even greater possibilities for their use in solving complex challenges in both established and cutting-edge fields.

### 4.1. Cyclodextrins in Nanomaterials and Nanostructured Coatings

The incorporation of cyclodextrins into nanomaterials and nanostructured coatings represents a groundbreaking development in material science. Nanomaterials, characterized by their exceptionally small size and high surface area, exhibit enhanced chemical reactivity, mechanical strength, and other advantageous properties that can be further improved with the inclusion of CDs. Cyclodextrins are utilized in various types of nanomaterials, including nanoparticles, nanofibers, and nanocomposites, to impart functional enhancements tailored to specific applications.

Cyclodextrin-modified nanoparticles are one of the most prominent applications in this domain, particularly in drug delivery systems. CDs are grafted onto the surface of nanoparticles to increase their drug-loading capacity and improve the stability of encapsulated drugs. For instance, β-cyclodextrin-functionalized gold nanoparticles have been explored for targeted drug delivery in cancer treatment [86,87]. The γ-CD’s hydrophobic cavity encapsulates anticancer drugs, such as doxorubicin, ensuring that the drug is protected from premature degradation and is delivered specifically to cancerous cells [88]. The result is a more efficient and targeted therapeutic approach, minimizing side effects and enhancing the drug’s efficacy.

In particular, polyamine and polyamide derivatives of cyclodextrins have garnered attention for their enhanced performance as effective coating agents for nanoparticles and gene delivery systems due to their unique structural properties and versatility in functionalization. Polyamine-modified cyclodextrins are characterized by the incorporation of multiple amine groups, which facilitate electrostatic interactions with negatively charged nucleic acids. This characteristic not only increases the stability of gene delivery formulations but also significantly enhances cellular uptake, promoting efficient gene transfection and expression within target cells. Similarly, polyamide derivatives of cyclodextrins possess improved structural stability and can form robust complexes with therapeutic agents and genetic material. Their capacity to encapsulate drugs or genes enables controlled-release profiles, which are essential for maintaining therapeutic efficacy while minimizing side effects. Furthermore, the functionalization of cyclodextrins with polyamines or polyamides can also improve targeting capabilities by allowing conjugation with ligands that recognize specific cell surface receptors, thereby increasing the precision of drug delivery [87].

Nanofibers, another type of nanomaterial, have benefited from cyclodextrin incorporation to create functional textiles. Cyclodextrin-based nanofibers are fabricated for use in air and water filtration systems, where their large surface area and high porosity allow for efficient trapping of pollutants [89]. Moreover, CDs in nanofibers can adsorb organic contaminants, such as volatile organic compounds (VOCs), offering an environmentally friendly solution for air purification [90].

In the field of nanostructured coatings, cyclodextrins are being utilized to confer specialized functionalities, such as antimicrobial properties, controlled-release mechanisms, and self-cleaning surfaces [91]. These coatings are particularly useful in industries requiring long-lasting, responsive, and environmentally adaptive materials.

Cyclodextrin-based smart coatings can be engineered to release active agents in response to environmental stimuli, such as temperature, pH, or humidity. This makes them ideal for applications in healthcare and packaging, where controlled release of antimicrobial agents or preservatives is required. For example, cyclodextrin-loaded antimicrobial coatings are being developed for use in medical devices and on hospital surfaces to prevent the spread of infections. These coatings can release antimicrobial agents gradually over time, reducing the risk of microbial colonization and improving sanitation.

In the domain of protective surfaces, cyclodextrin-based nano-coatings with self-cleaning properties are gaining attraction. These coatings, when exposed to sunlight or rain, can degrade contaminants, such as dust, oils, and even microorganisms, maintaining clean surfaces in outdoor environments. Such coatings are increasingly used on building materials, automotive surfaces, and even electronic devices to ensure longevity and aesthetic appeal.

Another material class of compounds represented by cyclodextrin-based nano-sponges can vary significantly in function. For example, carbonyl cross-linked nano-sponges, often made with agents such as diphenyl carbonate, excel in encapsulating hydrophobic drugs due to their stability and resistance to hydrolysis [92]. Epoxide cross-linked nano-sponges, such as those made with ethylene glycol diglycidyl ether, are more hydrophilic, making them suitable for drug delivery in aqueous environments and environmental applications [42]. Additionally, ionic functionalization introduces charged groups, enhancing their affinity for gene delivery applications through electrostatic interactions, while hydrophobic modifications improve the encapsulation of nonpolar compounds, aiding in drug solubility enhancement and pollutant removal. Polyamine-functionalized nano-sponges also show promise in gene delivery and stabilizing proteins due to their affinity for negatively charged biomolecules [93]. Biodegradable nano-sponges, created with cross-linkers such as citric acid, provide safe degradation in biological or environmental settings, making them especially valuable for pharmaceuticals requiring natural breakdown. Collectively, these diverse types of cyclodextrin-based nano-sponge materials expand the utility of cyclodextrins in fields such as targeted drug delivery, biomedicine, and environmental remediation, offering customizable properties to meet specific application needs.

### 4.2. Cyclodextrins in Environmental Remediation

Cyclodextrins also play a pivotal role in environmental remediation, particularly in addressing the growing concerns of pollution and the sustainable management of natural resources. Their ability to form inclusion complexes with hydrophobic and nonpolar molecules makes them effective in the removal of organic pollutants, such as polycyclic aromatic hydrocarbons (PAHs), pesticides, and dyes from contaminated environments [94,95].

Cyclodextrins, especially β-cyclodextrin, have been widely studied for the removal of organic pollutants from water and soil [96]. Their hydrophobic cavities can trap and isolate harmful substances, reducing their bioavailability and facilitating removal. In the case of polycyclic aromatic hydrocarbons (PAHs)—highly toxic organic pollutants that result from incomplete combustion—cyclodextrin complexes can solubilize these compounds, making them more accessible for degradation by microorganisms. This enhances the overall efficiency of bioremediation processes, helping to restore contaminated sites more quickly and effectively.

Cyclodextrins have also been successfully used to extract pesticides from agricultural soils and water systems. For example, α-cyclodextrin has been found effective in encapsulating atrazine, a commonly used herbicide that contaminates water sources [97]. When grafted onto cellulose, β-CD exhibits improved binding properties and stability, creating a versatile sorbent material that can selectively capture and remove pesticide residues from contaminated matrices. The complexation with cyclodextrin facilitates the removal of atrazine from the environment, reducing its negative impact on ecosystems and human health. This process not only removes the pesticide but also reduces its potential to leach into groundwater, thereby protecting water resources.

Beyond pollutant extraction, cyclodextrins are also being developed as carriers for the controlled release of agrochemicals, including pesticides and fertilizers. By encapsulating these chemicals within CDs, it is possible to release them more slowly and steadily, reducing environmental damage and increasing the efficacy of the chemicals. This controlled-release mechanism helps minimize over-application and runoff, which are major contributors to soil and water pollution.

For instance, cyclodextrin-based formulations of slow-release fertilizers are being developed to improve nutrient availability to plants, while minimizing leaching into groundwater [98]. This not only enhances crop productivity but also reduces the environmental footprint of agricultural activities. The use of CDs in these formulations allows for the gradual release of nutrients, such as nitrogen and phosphorus, preventing the rapid depletion of these essential elements and improving long-term soil health.

In addition to organic pollutants, cyclodextrins are also being explored for the remediation of heavy metal contamination. Cyclodextrins can be modified to selectively bind metal ions, such as lead, cadmium, and mercury, facilitating their removal from contaminated sites. For example, carboxymethyl-β-cyclodextrin has shown the ability to chelate toxic metals, allowing for their recovery or immobilization in soil and water systems [99]. This is particularly relevant for cleaning up industrial waste sites and mining areas where heavy metal contamination poses a significant threat to environmental and human health.

Cyclodextrin-based materials in environmental remediation provide an eco-friendly alternative to conventional methods, often involving harsh chemicals or energy-intensive processes. Their use helps reduce the overall environmental impact while improving the efficiency of pollution removal and resource recovery.

Volatile organic compounds (VOCs) pose significant environmental and health challenges, contributing to air pollution, smog formation, and respiratory issues. Their emissions arise from various sources, including industrial processes, vehicle exhaust, and household products, such as paints, solvents, and cleaning agents. The ability of cyclodextrins (CDs) to form inclusion complexes provides a viable solution for reducing VOC emissions across multiple applications. Cyclodextrins can encapsulate VOCs, effectively lowering their vapor pressure and enhancing their stability, which minimizes their release into the atmosphere.

In summary, the industrial applications of cyclodextrins are vast and diverse, with their role in nanomaterials and environmental remediation showcasing their multifunctional capabilities. By incorporating CDs into nanomaterials, industries can enhance product functionality, from drug delivery systems to smart coatings. Similarly, their ability to trap and release pollutants and agrochemicals in a controlled manner makes them invaluable tools for environmental sustainability.

## 5. Toxicity and Safety Considerations

The increasing utilization of cyclodextrins (CDs) across pharmaceuticals, food, environmental remediation, and other industries highlights the importance of thoroughly understanding their toxicity and safety profiles. While cyclodextrins are generally recognized as safe, particularly for oral administration, their safety is highly dependent on several factors, including the type of cyclodextrin, route of administration, dosage, and the guest molecules with which they form complexes. As cyclodextrins are integrated into more complex formulations, especially in the pharmaceutical and biomedical sectors, a comprehensive assessment of their toxicological profiles becomes essential.

### 5.1. Toxicological Profile of Cyclodextrins

Cyclodextrins, particularly the naturally occurring forms, such as α-, β-, and γ-cyclodextrin, have a generally favorable safety profile, especially when administered orally. For oral administration, native cyclodextrins tend to have low toxicity, as they are poorly absorbed in the gastrointestinal tract and are primarily excreted unchanged in the feces. For instance, γ-cyclodextrin has been granted generally recognized as safe (GRAS) status by regulatory agencies for use in food products and pharmaceuticals [100].

However, when cyclodextrins are administered via other routes, such as parenterally (e.g., intravenous or intramuscular injection), toxicity concerns may arise. β-Cyclodextrin, in particular, has been associated with nephrotoxicity when administered parenterally, especially at high doses [101]. This toxicity is largely due to its accumulation in the kidneys, where it can cause damage to renal cells. This is a major limitation for the use of β-cyclodextrin in parenteral drug formulations, necessitating the development and use of safer derivatives, such as hydroxypropyl-β-cyclodextrin (HP-β-CD), which have lower toxicity and better renal tolerance [102].

The degree of substitution in cyclodextrin derivatives also plays a crucial role in their toxicity. Substituted cyclodextrins, such as methylated CDs (e.g., randomly methylated β-cyclodextrin (RM-β-CD)) and hydroxypropylated CDs (e.g., HP-β-CD), exhibit varying degrees of toxicity depending on the nature and extent of the substitution [103]. For example, methylated cyclodextrins are known to interact with cell membranes more strongly than native CDs, which can lead to membrane disruption and cytotoxicity. This cytotoxic effect is particularly concerning for applications involving direct contact with cells or tissues, such as drug delivery systems or topical formulations [104]. Therefore, the selection of cyclodextrin derivatives for pharmaceutical or industrial applications must carefully balance their functional benefits with potential toxic effects.

Toxicity also depends on the specific cyclodextrin being used. α-Cyclodextrin is considered less toxic than β-cyclodextrin when administered orally. Research on the oral toxicity of α-cyclodextrin has shown that it is well tolerated in humans even at high doses, and it has been incorporated into food products as a dietary fiber [105].

### 5.2. Toxicity of Cyclodextrin Inclusion Complexes

The toxicity of cyclodextrins is not only dependent on the cyclodextrin molecule itself but also on the nature of the guest molecules with which they form inclusion complexes. The increase in bioavailability of guest molecules can also raise the risk of drug toxicity. For example, cyclodextrins can significantly increase the absorption of a drug by keeping it in a solubilized state, but if the dose is not properly adjusted, this can lead to drug concentrations that exceed therapeutic thresholds, resulting in toxic effects [106].

The pharmacokinetics of a drug can also be significantly altered by complexation with cyclodextrins. For instance, drugs with narrow therapeutic windows, such as certain chemotherapeutic agents, may pose a higher risk of toxicity when their bioavailability is enhanced by cyclodextrin inclusion complexes. Cyclodextrins can also alter the distribution of drugs within the body, potentially leading to increased accumulation in specific tissues, thereby exacerbating drug-induced toxicity in those areas [107].

Additionally, the release kinetics of the guest molecule from the cyclodextrin complex can be affected by environmental factors, such as pH, temperature, and the presence of other competing substances. In drug delivery systems, this variability in release kinetics can complicate the dosing and predictability of therapeutic effects. For example, in acidic environments, such as the stomach, the inclusion complex may dissociate faster, leading to a rapid release of the drug, which could cause irritation or other local toxic effects. Conversely, in more neutral or basic environments, the drug may be released more slowly, potentially delaying therapeutic effects or causing uneven drug distribution [108].

In industrial applications, such as environmental remediation, where CDs are used to trap pollutants or facilitate the release of agrochemicals, the potential toxicity of both the cyclodextrin and the guest molecule must be carefully evaluated. While CDs can reduce the environmental mobility of pollutants by encapsulating them, the complexation process may also alter the behavior of the pollutants, affecting their degradation or bioaccumulation potential. For example, the use of CDs to capture pesticides may reduce their immediate environmental toxicity but could also slow down their breakdown, leading to long-term persistence in the environment [109].

Comprehensive in vitro and in vivo studies are necessary to fully assess the toxicity of cyclodextrin-based formulations, particularly those that involve new or highly modified cyclodextrin derivatives. Toxicological studies should include evaluations of the safety of both the cyclodextrin and the inclusion complex, considering factors such as tissue distribution, metabolic stability, and excretion pathways. Regulatory agencies, such as the FDA and EMA, require extensive safety data before approving cyclodextrin-containing drugs, especially for routes of administration other than oral. As CDs continue to be incorporated into more sophisticated drug delivery systems and industrial applications, it is critical to maintain stringent safety evaluations to mitigate potential risks.

In conclusion, while cyclodextrins have established themselves as safe and effective excipients for many applications, especially in drug delivery and food industries, their toxicological profiles must be carefully considered, particularly in more sensitive routes of administration, such as parenteral delivery. The type of cyclodextrin, its degree of substitution, and the nature of the inclusion complex all play crucial roles in determining safety. Through continued research and rigorous testing, the safe and effective use of cyclodextrins in diverse fields can be ensured, balancing their beneficial properties with any potential risks.

## 6. Future Prospects

The future of cyclodextrin (CD) research and applications is poised to expand significantly as new advancements in chemistry, biotechnology, and material science continue to emerge. The unique properties of CDs, coupled with the growing ability to design and synthesize novel derivatives, offer tremendous opportunities for innovation across various fields.

### 6.1. Advances in Cyclodextrin Chemistry and Derivative Design

The development of new cyclodextrin derivatives with enhanced and tunable properties will remain a key area of focus. Emerging synthetic techniques, such as site-specific functionalization and green chemistry approaches, will likely lead to the creation of more selective and efficient CDs. These advances could result in derivatives with improved complexation capacities, greater biocompatibility, and targeted release profiles. Furthermore, the application of computational chemistry and machine learning in the design of CD derivatives could accelerate the discovery of novel structures with optimized performance for specific applications. Understanding the molecular interactions within cyclodextrin inclusion complexes is essential for optimizing their design and applications across various fields. While experimental techniques provide direct insights into the structural and physicochemical properties of these complexes, computational methods, such as quantum chemical calculations and molecular dynamics (MD) simulations, have become increasingly important for studying the detailed interactions between cyclodextrins and their guest molecules. Quantum chemical calculations involve the use of methods, such as density functional theory (DFT), to model the electronic structure and interaction energies of cyclodextrin inclusion complexes [110]. These calculations can predict binding energies, charge distribution, and molecular orbitals, offering a deeper understanding of the forces that govern the complexation process. By simulating the interactions between the cyclodextrin cavity and the encapsulated guest at an atomic level, quantum chemical calculations can reveal hydrogen bonding patterns, van der Waals interactions, and dipole–dipole interactions that contribute to the stability and selectivity of the inclusion complex. Molecular dynamics (MD) simulations are another powerful tool for studying the dynamic behavior of cyclodextrin inclusion complexes over time [111]. MD simulations allow researchers to visualize how guest molecules interact with cyclodextrins in different solvent environments and under various temperature and pressure conditions. Through these simulations, it is possible to observe guest encapsulation and release mechanisms, rotational and translational motions, and the structural flexibility of the cyclodextrin ring. Combining quantum chemical calculations and MD simulations with experimental techniques allows for a more holistic understanding of cyclodextrin inclusion complexes. Computational studies can predict binding affinities and structural configurations that can be verified through methods such as NMR spectroscopy and X-ray crystallography, while experimental data can validate and refine the models used in simulations. The ongoing development of customizable cyclodextrins using AI-driven design could lead to more efficient and selective drug delivery systems, particularly for complex biologics [112].

### 6.2. Cyclodextrins in Drug Delivery and Therapeutics

In the pharmaceutical sector, the role of cyclodextrins is expected to expand as drug delivery systems become more sophisticated. The development of CDs for the delivery of biologics, such as peptides, proteins, and nucleic acids, is an exciting frontier. These macromolecules often face challenges related to stability and delivery, which CDs could help address through protective encapsulation and targeted release mechanisms. Additionally, the integration of CDs into nanocarriers, such as liposomes and polymeric nanoparticles, could further enhance the efficacy and safety of drug delivery systems. Personalized medicine is another area where CDs could play a crucial role. The ability to tailor CD-based formulations to the individual needs of patients, considering factors such as the genetic profile, disease state, and pharmacokinetics, could lead to more effective and safer therapeutic strategies. Advances in drug delivery technology, such as responsive or stimuli-sensitive CDs, may allow for more precise control over drug release, further optimizing treatment outcomes [113].

### 6.3. Environmental and Industrial Applications

Cyclodextrins are expected to have a growing impact on environmental and industrial processes, particularly in the context of sustainability. The use of CDs in environmental remediation will likely expand as concerns about pollution and resource conservation intensify. New CD-based materials designed for the capture and degradation of emerging pollutants, such as microplastics and pharmaceutical residues, are likely to emerge. Moreover, CDs could play a role in the circular economy by facilitating the recovery and reuse of valuable resources from waste streams. In industrial applications, CDs will continue to be explored as additives and functional materials in areas such as food preservation, cosmetics, and textiles. The development of CD-based smart materials—capable of responding to environmental changes—could revolutionize sectors such as packaging, where such materials could extend the product’s shelf life and reduce waste. Additionally, CDs may become integral to the development of more sustainable manufacturing processes, where their ability to form inclusion complexes could be harnessed for the selective extraction, purification, or stabilization of industrial products [114].

### 6.4. Safety and Toxicology Research

As the application of CDs broadens, the need for comprehensive safety and toxicology research will be more pressing. Advances in in vitro and in silico methods, including high-throughput screening and predictive toxicology, will likely play a significant role in assessing the safety of new CD derivatives and formulations. Understanding the long-term effects of CDs, particularly in chronic use or environmental exposure, will be critical. Collaborative efforts between industry, academia, and regulatory bodies will be essential in establishing guidelines and safety standards for the use of CDs in various applications.

### 6.5. Cyclodextrins in Emerging Technologies

Cyclodextrins may also find new roles in emerging technologies, such as biotechnology, artificial intelligence, and renewable energy. In biotechnology, CDs could be used to stabilize or enhance the performance of enzymes and other biocatalysts, improving the efficiency of industrial bioprocesses. In renewable energy, CDs could contribute to the development of novel storage materials, such as batteries and supercapacitors, by improving the stability and performance of active materials. Moreover, the intersection of CDs with artificial intelligence and machine learning could lead to the discovery of new applications by predicting complexation behavior, optimizing synthesis pathways, and identifying new functional materials. This cross-disciplinary approach could significantly accelerate the innovation cycle, leading to faster development and deployment of CD-based technologies.

## 7. Conclusions

Cyclodextrins represent a versatile class of molecules with wide-ranging applications in chemistry, biology, and industry. Their ability to form inclusion complexes with various guest molecules has made them indispensable in pharmaceuticals, environmental remediation, and material science. The development of cyclodextrin derivatives has further expanded their utility, allowing for the customization of their properties to suit specific applications. However, the growing use of CDs also underscores the importance of understanding their toxicological profile. While many CDs are considered safe for use, particularly in low concentrations and with proper formulation, the potential for toxicity, especially with certain derivatives and inclusion complexes, requires careful consideration. As research continues to advance, the development of safer and more effective CD-based systems will be crucial in unlocking their full potential across diverse fields.

This review highlighted the multifaceted nature of cyclodextrins and underscored the need for ongoing research to optimize their applications while ensuring their safety. By bridging the gap between chemistry, biology, and industry, cyclodextrins have the potential to drive innovation and address some of the most pressing challenges in modern science and technology.

Cyclodextrins have already demonstrated their versatility and impact across multiple disciplines, from pharmaceuticals to environmental science. Looking ahead, the continued exploration and development of CD derivatives, coupled with advances in technology and a growing emphasis on sustainability, will likely unlock new applications and enhance existing ones. As research progresses, the potential for CDs to address emerging challenges and contribute to innovative solutions is immense. The future of cyclodextrins is bright, with the promise of significant contributions to science, technology, and society at large.

## Figures and Tables

**Table 1:** The most important characteristics of α-CD, β-CD, and γ-CD [5].

Property	Alpha	Beta	Gamma
Glucose subunits Synonyms Height	Hexa Cyclo-hexaamylose (alfadex) 7.9	Hepta Cyclo-heptaamylose (betadex) 7.9	Octa Cyclo-octaamylose (gammadex) 7.9
Cavity diameter	4.5–5.3	6–6.5	7.5–8.3
External diameter	14.6	15.4	17.5
Molecular weight	972	1135	1297

**Table 2:** Comparison of cyclodextrin derivatives and their properties.

Cyclodextrin Derivative	Solubility (mg/100 mL)	Toxicity (LD_50_ of NOEL Values)	Applications	References
α-Cyclodextrin (α-CD)	14.5	Low (1000 mg/kg; rat; IV route)	Food additives, pharmaceuticals	[27]
β-Cyclodextrin (β-CD)	1.8	Moderate (nephrotoxicity) (788 mg/kg; rat; IV route)	Pharmaceuticals, environmental remediation	[28,29]
γ-Cyclodextrin (γ-CD)	23.2	Low (>3750 mg/kg; rat; IV route)	Drug delivery, food industry	[18]
Hydroxypropyl-β-cyclodextrin (HP-β-CD)	>60.0	Very Low (NOEL: 500 mg/kg/day; oral)	Drug solubilization, cosmetics	[30]
Methyl-β-cyclodextrin (M-β-CD)	50.0	High (cytotoxicity) (>8000 mg/kg; rat; oral route)	Research, drug delivery	[28]
Sulfobutylether-β-cyclodextrin (SBE-β-CD)	>50.0	Low (NOEL: 500 mg/kg/day; oral)	Injectable drug formulations, toxicity reduction	[31]

**Table 3:** Recent applications of some cyclodextrins in drug formulations.

Drug	Cyclodextrin Type	Application	Benefit	Example of Effect
Griseofulvin	β-Cyclodextrin	Antifungal therapy	Increased solubility and improved absorption	Enhanced bioavailability leading to effective treatment [67]
Ibuprofen	β-Cyclodextrin	Pain relief	Enhanced absorption rates	Faster onset of action and improved therapeutic effect [68]
Digoxin	2-Hydroxypropyl-β-cyclodextrin	Cardiac therapy	Prolonged duration of action	Reduced dosing frequency and stabilized plasma levels [69]
Erythromycin	Hydroxypropyl-β-cyclodextrin	Antibiotic treatment	Enhanced stability in acidic environments	Maintained drug efficacy following oral administration [70]
Curcumin	β-Cyclodextrin	Anti-inflammatory	Improved solubility and bioavailability	Greater therapeutic effects in managing inflammation [71]
Theophylline	Methylated β-cyclodextrin	Asthma treatment	Controlled release and reduced side effects	Steady plasma concentrations resulting in effective therapy [72]
Taxol (Paclitaxel)	2-Hydroxypropyl-β-cyclodextrin	Cancer treatment	Enhanced solubility and bioavailability	Improved therapeutic index and reduced toxicity [73]

**Table 4:** Applications of cyclodextrin inclusion complexes across various industries and their functional benefits.

Application Field	Guest Molecule	Cyclodextrin Type	Function/Benefit	Example
Pharmaceuticals	Poorly soluble drugs (e.g., antifungals, steroids)	β-CD, γ-CD	Enhances solubility, bioavailability, and stability of drugs	Improved delivery of drugs, such as itraconazole and dexamethasone [76]
Food industry	Flavors, essential oils, vitamins	α-CD, β-CD, γ-CD	Stabilizes volatile compounds, protects against oxidation, improves shelf life	Encapsulation of limonene (flavor) to prevent evaporation in citrus-based products [77]
Cosmetics and personal care	Fragrances, vitamins (e.g., retinol), peptides	β-CD, modified CDs	Controls release of active ingredients, enhances stability of sensitive compounds, reduces skin irritation	Controlled release of fragrances in perfumes or sustained release of retinol in anti-aging creams [78]
Environmental remediation	Organic pollutants (e.g., pesticides, hydrocarbons)	β-CD, modified CDs	Traps and removes organic pollutants from water and soil, reduces environmental impact	Use of CD-based materials for removing phenols or pesticides from contaminated water [79]
Agriculture	Pesticides, herbicides, plant growth regulators	β-CD, γ-CD	Improves solubility and stability of agrochemicals, reduces environmental toxicity	Cyclodextrin-enhanced delivery of herbicides, reducing runoff and improving soil uptake [80]
Biomedical	Anticancer agents, gene therapy vectors	β-CD, γ-CD, modified CDs	Enhances targeted drug delivery, improves the stability and release of biomolecules	Cyclodextrin-based nanocarriers for delivering doxorubicin in cancer therapy [81]
Food safety analysis	Contaminants (e.g., toxins, preservatives, pesticides)	α-CD, β-CD	Enhances sensitivity and accuracy of detecting harmful substances in food	Detection of pesticide residues and mycotoxins in food products [82]
Textile industry	Fragrances, antimicrobial agents	β-CD, modified CDs	Encapsulation for controlled release of fragrances or antimicrobial agents in fabrics	CDs used in fabrics to provide long-lasting fragrance release or antimicrobial properties [83]
Nanotechnology	Nanoparticles, drugs, bioactive molecules	β-CD, γ-CD, modified CDs	Enables formation of nanocarriers for targeted delivery and diagnostic purposes	Cyclodextrin-based nanoparticles for targeted drug delivery in cancer treatment or imaging [84]
Analytical chemistry	Chiral molecules, toxins	β-CD, modified CDs	Used in chromatographic separations and sensing for chiral or hazardous compounds	Chiral separation in high-performance liquid chromatography (HPLC) using cyclodextrin-modified columns [85]
Packaging	Oxygen, ethylene gas	β-CD, γ-CD	Absorbs gases to extend the shelf life of packaged products, prevents spoilage	Cyclodextrins used in packaging materials to absorb ethylene gas in fresh produce packaging, extending shelf life [15]
